# Effects of Salt Stress on the Morphology, Growth and Physiological Parameters of *Juglans* *microcarpa* L. Seedlings

**DOI:** 10.3390/plants11182381

**Published:** 2022-09-12

**Authors:** Xinying Ji, Jiali Tang, Junpei Zhang

**Affiliations:** State Key Laboratory of Tree Genetics and Breeding, Key Laboratory of Tree Breeding and Cultivation of the State Forestry and Grassland Administration, Research Institute of Forestry, Chinese Academy of Forestry, Beijing 100091, China

**Keywords:** *Juglans microcarpa* L., rootstock, salt stress, growth characteristics, physiological index

## Abstract

In this study, to screen for walnut salt-tolerant rootstocks, *Juglans microcarpa* L. seedlings were treated in different NaCl concentrations (0, 50, 100, 200, and 300 mmol/L), and the growth situation of seedlings was observed. Moreover, we determined the physiological indexes of seedlings on different days (6, 12, 18, and 24 d) after treatment. The results showed that after salt stress, the external morphology of seedlings displayed salt injury, which manifested as yellowing, withering, curling, and falling off of leaves. High concentrations and long-term stress led to more serious damage, with numerous leaves undergoing withering and shedding. Salt stress significantly inhibited the growth of seedlings. With the increase in salt concentration and stress time, the chlorophyll content and photosynthetic parameters of seedlings reduced to varying degrees; the relative electrical conductivity (REC) and malondialdehyde (MDA) increased. Superoxide dismutase (SOD), peroxidase (POD), and catalase (CAT) activities generally increased, followed by a decrease; proline (Pro) accumulated; and soluble sugar (SS) content first increased and then decreased. In addition, it promoted the production of abscisic acid (ABA) and inhibited the synthesis of indole-3-acetic acid (IAA), gibberellic acid 3 (GA3), and zeatin riboside (ZR). It was found that *J.* *microcarpa* L. seedlings were more tolerant under 100 mmol/L salt stress, whereas the damage to growth was more severe at 200 mmol/L to 300 mmol/L salt stress.

## 1. Introduction

Currently, soil salinization is a worldwide resource and environmental problem. Due to unreasonable irrigation, misuse of chemical fertilizers, and industrial pollution, the salinization problem is becoming increasingly prominent, thus severely restricting the sustainable development of agriculture and forestry [1]. On a worldwide scale, 20% of arable lands and 50% of irrigated lands are affected by salinity [2]. According to statistics, the total area of different types of saline soils in China is approximately 350,000 km^2^, accounting for about 25% of the total arable land area. The six western provinces, three northeastern provinces, and the lower terrain areas in the coastal provinces of China are areas where saline soils are abundantly present [3,4]. The vast saline soil is considered an important reserve land resource in China. The rational development and use of saline soil is of great significance for the ecological environmental protection and sustainable development of China’s agriculture and forestry economy.

Sodium chloride (NaCl) is the most plentiful salt that is conducive to soil salinization. Exposure of plants to salt stress causes a series of growth, physiological, and biochemical changes, and the effects of salinity on plant growth and physiology are deleterious and complicated, largely due to the physiological and metabolic activities of plants impaired by osmotic stress, ionic stress, and nutritional imbalances or a combination of these factors [5,6]. Under high soil salinity conditions, excessive soluble Na^+^ and Cl^−^ increase the osmotic potential of the soil solution, thus inhibiting root water uptake, reducing leaf expansion and stomatal closure, and restricting the photosynthesis and respiration in plants [5,7]. As mineral elements, Na^+^ and Cl^−^ are essential for the normal growth and development of plants. However, if absorbed by the root system in large amounts, they will accumulate in the leaves and cause toxicity to cell metabolism and the function of some enzymes. Finally, they can lead to premature senescence of leaves and other negative effects, including impaired protein synthesis, reactive oxygen species (ROS) production, and disturbances in energy and lipid metabolism [5].

Currently, the research on plant salt tolerance is largely focused on external morphological characteristics [8,9], osmoregulation [5,10], antioxidant protection [11,12], ion homeostasis [9,10], anatomical structure [13,14], hormonal regulation [15,16], and intrinsic molecular regulatory mechanisms [17,18]. To investigate the changes in plant growth and physiological and biochemical characteristics under salt stress is an important basis for selecting and breeding salt-tolerant or salt-resistant plant materials. In addition, it is an economical and effective method to improve the use of saline–alkali lands.

*J. microcarpa* L. belongs to the genus *Juglans* of the Juglandaceae family; it is a type of black walnut that is native to the southwestern United States and is recognized as a dwarf rootstock for cultivating excellent clones of walnut and black walnut. *J. microcarpa* L. has a large canopy and a beautiful posture, making it a good tree species for urban street beautification [3,19]. It was introduced to China from the United States by Mr. Xi Shengke and others in 1984 and has been planted in Henan, Inner Mongolia and other places [3]. At present, only a few studies exist on the salt tolerance of *J. microcarpa* L. Therefore, in this study, we explored the salt tolerance of *J. microcarpa* L. using its current-year seedlings as the experimental material. We investigated the dynamic changes in the external morphology, growth, and physiological indexes of *J. microcarpa* L. under different concentrations of NaCl treatment and different stress times and hope to provide a theoretical basis for the screening of salt-tolerant rootstocks of walnut.

## 2. Results

### 2.1. Effects of Salt Stress on External Morphological Characteristics of Seedlings

#### 2.1.1. Symptom Classification of Salt Injury

The changes in the external morphology of plants under each treatment were observed and recorded at 6, 12, 18, and 24 days after salt stress, respectively. The symptoms of salt damage reflected by the external morphology of *J. microcarpa* L. seedlings were graded according to the overall plant condition and leaf damage symptoms with reference to the description provided by Wang Xiaoli [20], as shown in Table 1.

#### 2.1.2. Change in Morphological Characteristics

The degree of plant stress at each concentration at different stress periods was recorded (Table 2). At the early stage of stress (6 days), there was no significant change in each treatment. At 12 days of salt stress, under 200 mmol/L NaCl treatment, the leaves in the lower part of the plant were yellowed, the leaf tips were withered, and the number was less. Under 300 mmol/L NaCl treatment, the lower old leaves were partially yellowed, and the leaf tips and margins were withered. At 18 days of salt stress, all treatment concentrations showed different degrees of salt damage symptoms, and the lower leaves of the plants showed withered symptoms at 50 to100 mmol/L concentration; under 200 mmol/L concentration, the lower leaves of the plant withered more deeply and in greater numbers, and accompanied by leaf curling, the upper leaves showed a certain degree of leaf margin withering; under 300 mmol/L concentration, the withering and curling of the leaves of the whole plant increased, especially the lower leaves showed more withering and curling, and the lower compound leaves and the total petiole showed drying and abscission. The leaf damage symptoms increased significantly at 24 days of salt stress, and the number of withered and curled leaves increased at 50 mmol/L and 100 mmol/L concentrations. At a NaCl concentration of 200 mmol/L, the number of withered leaves of the whole plant was higher, the area of withered leaves was larger, the curling was severe, and the compound leaves and total petioles of the lower part of the plant dried up and fell off to a small amount. At a NaCl concentration of 300 mmol/L, the withered leaves and the curly degree of the leaves were further deepened, and the compound leaves and total petioles of the whole plant dried up and fell off greatly.

### 2.2. Effects of Salt Stress on Plant Growth

The effects of salt stress on growth indexes of *J. microcarpa* L. are shown in Table 3. With an increase in the NaCl concentration, *H*_Δ_ and *D*_Δ_ of *J. microcarpa* L. showed a decreasing trend. When the concentration of NaCl reached 200 mmol/L, *H*_Δ_ reached a significant difference compared with the control, with a decrease of 44.13%. At 300 mmol/L, *D*_Δ_ decreased by 35.83% compared with the control, with no significant difference among the treatments.

With an increase in the NaCl concentration, DWS and PDW of *J. microcarpa* L. exhibited a declining trend, resulting in a significant difference with the control at 200 mmol/L and a decrease ratio of 40.06% and 28.59%, respectively. At 50 mmol/L, DWR increased compared with the control; however, the difference was insignificant, and as the NaCl concentration continued to increase, the DWR decreased gradually and decreased by 38.11% at 300 mmol/L, which was significantly different from the control. R/S reflects the relationship between the aboveground and underground parts of plants under stress. The overall trend of R/S increased at NaCl concentrations of less than 200 mmol/L, reaching a maximum at 200 mmol/L with an increase of 45.96%, followed by a decreased at 300 mmol/L.

### 2.3. Effects of Salt Stress on Chlorophyll Content

**Figure 1:**
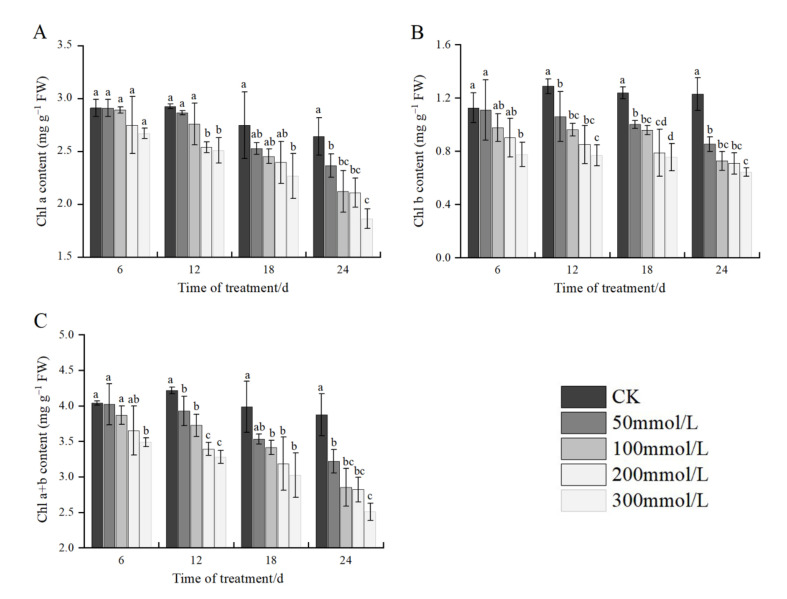
The effect of salt stress on chlorophyll content of *J. microcarpa* L. seedlings. (**A**) Chlorophyll a content (Chl a), (**B**) chlorophyll b content (Chl b), and (**C**) total chlorophyll content (Chl a + b) in the leaves. Error bars indicate the SD of treatment means. Different letters on the bars indicate significant differences among the treatments (*p* < 0.05).

As shown in Figure 1, the content of Chl a, Chl b, and Chl a+b in the leaves of *J. microcarpa* L. declined with an increase in the NaCl concentration at the same stress time. At 6 days of salt stress, compared with the control, the content of Chl a, Chl b, and Chl a+b decreased by 8.27%, 31.17%, and 14.63%, respectively, where Chl a content was not significantly different among treatments, and Chl b and Chl a+b contents were significantly different from that of the control at 300 mmol/L. At 12 days of salt stress, the content of Chl a, Chl b, and Chl a+b decreased by 14.25%, 40.30%, and 22.20%, respectively, compared with the control. At 18 days of salt stress, the content of Chl a, Chl b, and Chl a+b decreased by 17.45%, 38.98%, and 24.12%, respectively, compared with the control. At 24 days of salt stress, the above indices were significantly different among the treatments, which decreased by 29.44%, 47.68%, and 35.23%, respectively, compared with the control.

Under the same salt concentration treatment, the content of Chl a, Chl b, and Chl a+b decreased with an increase in stress time at all salt concentrations, except the control, and decreased to the lowest after 24 days of salt stress, with the maximum decrease of 29.44%, 47.68%, and 35.23%, respectively. The above results showed that salt stress reduced the chlorophyll synthesis in *J. microcarpa* L., and the chlorophyll content decreased gradually with an increase in the salt concentration and the extension of stress time, in which the content of Chl b was the most affected by salt stress.

### 2.4. Effects of Salt Stress on Photosynthetic Parameters

**Figure 2:**
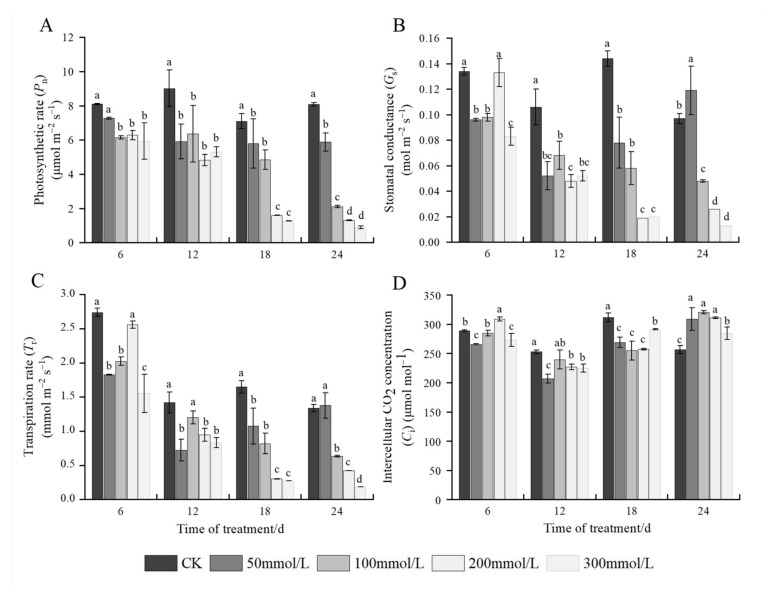
Effect of salt stress on the photosynthetic parameters of *J. microcarpa* L. seedlings. (**A**) Photosynthetic rate (*P*_n_), (**B**) stomatal conductance (*G*_s_), (**C**) transpiration (*T*_r_), and (**D**) intercellular CO_2_ concentration (*C*_i_) in the leaves. Error bars indicate the SD of treatment means. Different letters on the bars indicate significant differences among the treatments (*p* < 0.05).

As shown in Figure 2A, under the same time stress, the *P*_n_ of *J. microcarpa* L. decreased with an increase in the NaCl concentration. At 6 days, 18 days, and 24 days of salt stress, *P*_n_ reached the lowest value at 300 mmol/L, which decreased by 26.77%, 82.24%, and 89.09% compared with the control, respectively. At 12 days of salt stress, the decrease in *P*_n_ at 200 mmol/L was the largest, which was 46.42%; however, the difference with the value at 300 mmol/L was insignificant. At 18 days of salt stress, a significant difference was recorded between the treatments and the control, and *P*_n_ decreased more at salt concentrations of 200 mmol/L–300 mmol/L compared with the control, which was 77.52% and 82.24%, respectively. At 24 days of salt stress, the *P*_n_ decreased by 27.22%, 73.89%, 83.91%, and 89.09%, respectively, compared with the control, and all differences were significant. Under the same salt concentration treatment, the *P*_n_ in the control treatment did not differ significantly; *P*_n_ decreased with an increase in stress time under each salt concentration treatment. At 50 mmol/L, the *P*_n_ slowly decreased with an increase in stress time. At 100 mmol/L, the *P*_n_ declined rapidly on the 24th day, whereas at 200 mmol/L and 300 mmol/L, the *P*_n_ decreased rapidly on the 18th day and then changed slowly with time.

The *G*_s_ of *J. microcarpa* L. decreased significantly with an increase in the salt concentration. At the same stress time, the *G*_s_ significantly decreased by 38.06%, 54.72%, 86.81%, and 86.60% at 6 days, 12 days, 18 days, and 24 days of salt stress, respectively, compared with the control. Under the same salt concentration treatment, *G*_s_ showed a decreasing trend with increasing stress time, and when the stress time was longer than 18 days, the change in *G*_s_ under 100 mmol/L–300 mmol/L gradually slowed down (Figure 2B).

The trend of *T*_r_ changes in seedling leaves under salt stress was similar to that of *G*_s_. Under the same salt stress time, it decreased with an increase in the salt concentration, and at a salt concentration of 300 mmol/L, with decreases of 43.32%, 41.58%, 83.43%, and 86.23% compared with that of the control, respectively. *T*_r_ showed a decreasing trend with an increase in stress time under the same salt concentration treatment (Figure 2C).

At 6 days of salt stress, the *C*_i_ of *J. microcarpa* L. decreased initially, followed by an increase with the increase in salt stress concentration, and reached the lowest value at 50 mmol/L treatment. At 12 days of salt stress, the *C*_i_ showed a decrease with an increase in salt stress concentration. At 24 days of stress, *C*_i_ increased initially, followed by a decrease, and reached a maximum at a salt concentration of 100 mmol/L. Under the same salt concentration treatment, *C*_i_ decreased initially and subsequently increased with an increase in stress time, except for the salt concentration of 300 mmol/L, which reached its peak at 24 days of salt stress (Figure 2D).

### 2.5. Effects of Salt Stress on REC and MDA Content

**Figure 3:**
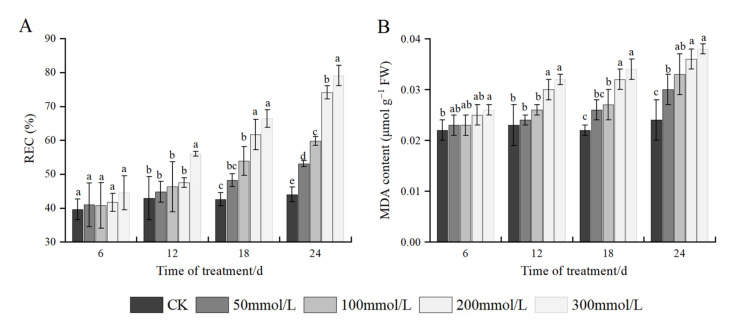
Effect of salt stress on (**A**) relative electrical conductivity (REC), (**B**) malondialdehyde (MDA) content of *J. microcarpa* L. seedlings. Error bars indicate the SD of treatment means. Different letters on bars indicate significant differences among the treatments (*p* < 0.05).

As shown in Figure 3, under the same time stress, the REC and MDA contents of *J. microcarpa* L. increased continuously with an increase in salt concentration. At 6 days of salt stress, a small change in REC was recorded among treatments; at 12 days of salt stress, the REC was significantly different from the control at 300 mmol/L, with an increase of 30.54%. At 24 days of salt stress, the REC showed a continuous upward trend, with significant differences among all treatments, and each salt concentration increased compared to the control by 20.68%, 35.91%, 68.41%, and 79.77% compared with the control. The trend of MDA content was roughly consistent with the change in REC: at 6 days of salt stress, the MDA content was highest at 300 mmol/L, with an increase of 18.18%. The maximum increase in MDA was 39.13% at 12 days of salt stress, 54.55% at 18 days of salt stress, and 58.33% at 24 days of salt stress. At the same salt concentration, the REC and MDA contents of seedling leaves increased with an increase in stress time and reached the maximum on the 24th day.

### 2.6. Effects of Salt Stress on Osmotic Adjustment Substances

**Figure 4:**
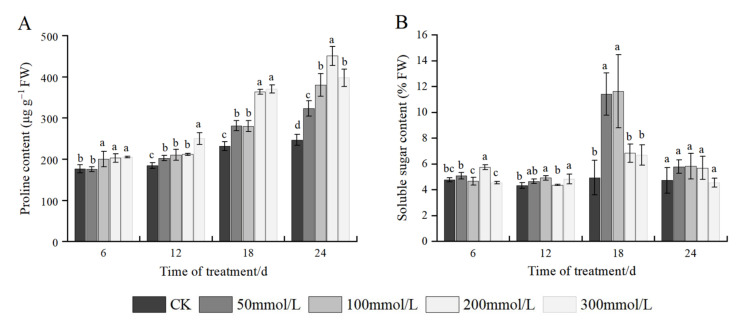
Effect of salt stress on osmotic adjustment substances of *J. microcarpa* L. seedlings. (**A**) Proline (Pro) content, (**B**) soluble sugar (SS) content in the leaves. Error bars indicate the SD of treatment means. Different letters on the bars indicate significant differences among the treatments (*p* < 0.05).

As shown in Figure 4A, under the same time stress, as the concentration of NaCl increased, the Pro content in seedlings also increased. At 6 days of salt stress, significant differences were observed, with concentrations ranging from 100 mmol/L to 300 mmol/L, with a maximum increase of 16.10% compared with the control. At 12 days of stress, all treatments were significantly different from the control, and the highest Pro content was found at 300 mmol/L with an increase of 35.76%. At 18 days of stress, the Pro content continued to increase, showing a significant difference between each treatment and the control, and at 200 mmol/L and 300 mmol/L, the content of Pro was similar, which increased by 57.15% and 60.08%, respectively, compared with the control. At 24 days of stress, each salt concentration increased by 31.17%, 54.25%, 82.83%, and 61.33%, respectively, compared with the control. In addition, at the same salt concentration, the Pro content also increased with an increase in stress time, with longer stress times leading to higher Pro content.

As shown in Figure 4B, at the same stress time, the difference in the SS content among the groups before treatment was insignificant. At 6 days of stress, the SS content increased at first, followed by a decrease and the highest SS content was recorded under 200 mmol/L, which was significantly different from the control, with no significant difference between other treatments and the control. At 12 days of stress, the SS content increased, followed by a decrease with an increase in the salt concentration, and the SS content under 100 mmol/L salt stress was significantly different from that of the control, and the content was the highest. At 18 days of stress, the content of SS increased at first, followed by a decrease with an increase in the salt concentration, and the SS content was significantly higher under 50 and 100 mmol/L salt stress, under 50 and 100 mmol/L salt stress, the content of SS was significantly higher than that of other treatments, and the SS content was the highest at 100 mmol/L, whereas the difference between other salt treatments and control was insignificant. At 24 days of stress, the SS content increased, followed by a decrease; however, there was no significant difference among the treatments, and the SS content was the highest at 100 mmol/L. At the same salt concentration, the SS content first increased and subsequently decreased as the stress time increased, and the peak SS content of all treatments was found on the 18th day after salt stress, which subsequently decreased significantly.

### 2.7. Effects of Salt Stress on Antioxidant Enzyme Activities

**Figure 5:**
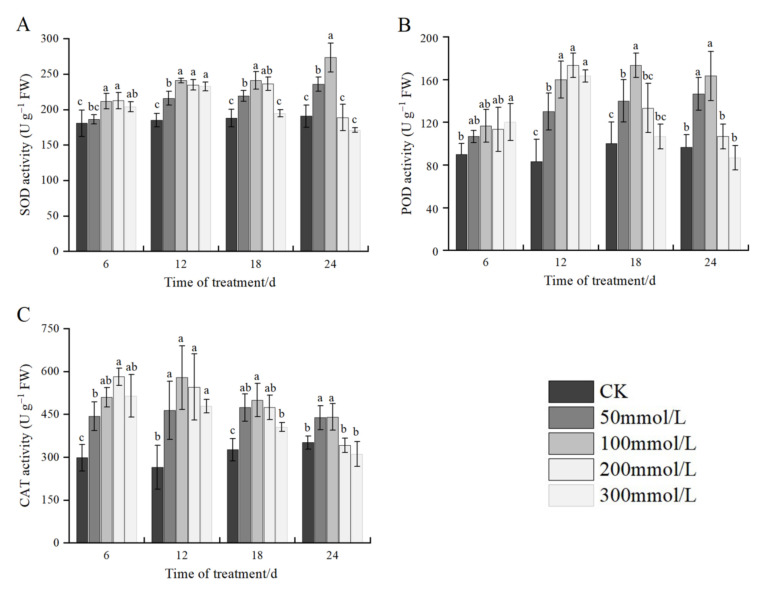
Effect of salt stress on the activities of antioxidant enzymes in leaves of *J. microcarpa* L. seedlings. (**A**) Superoxide dismutase (SOD) activity, (**B**) peroxidase (POD) activity, and (**C**) catalase (CAT) activity of leaves. Error bars indicate the SD of treatment means. Different letters on the bars indicate significant differences among the treatments (*p* < 0.05).

As shown in Figure 5A, at the same stress time, the SOD activity increased, followed by a decrease with an increase in NaCl concentration, reaching a maximum of 100 mmol/L. At 12 days of salt stress, the SOD activity increased the most at 100 mmol/L and 200 mmol/L compared with the control, with an increase of 30.18% and 26.87%, respectively. At 24 days of salt stress, the activity of SOD decreased compared with the control at 200 mmol/L and 300 mmol/L salt concentration; however, there was no significant difference from the control. Under the same concentration treatment, the SOD activity in the leaves showed a continuous increase with an increase in stress time at CK-100 mmol/L; the SOD activity increased at first and subsequently decreased from 200 mmol/L to 300 mmol/L, with peaks on the 18th and 12th days, respectively.

Under the same stress time, the POD activity was different under different NaCl concentrations. At 6 days of salt stress, the POD activity increased with an increase in NaCl concentration, and only the concentration of 300 mmol/L increased significantly compared with the control, with an increase of 33.33%. With the extension of stress time, the POD activity increased at first and then decreased. At the same salt stress level, with the extension of stress time, the POD activity showed an increasing trend at 50 mmol/L, whereas it first increased and subsequently decreased at 100 mmol/L–300 mmol/L, among these concentrations, 100 mmol/L treatment reached the maximum at 18 days of salt stress, and the other concentrations reached the peak at 12 days (Figure 5B).

At the same stress time, the trend in the CAT activity was similar to that of SOD, which increased at first and subsequently decreased with an increase in NaCl concentration. On the 6th day, the activity was the highest at 200 mmol/L. On the 12th, 18th, and 24th days after the treatment, the CAT activity reached the peak at a concentration of 100 mmol/L NaCl, with a significant difference between the control and treatments. At 24 days of stress, the CAT activity under 200 and 300 mmol/L showed a decrease compared with the control, however, the difference was insignificant. At the same level of salt stress, the CAT activity increased and subsequently decreased with an extension in stress time, and the CAT activity at 50 mmol/L concentration reached the peak after 18 days, and the other concentrations reached the maximum after 12 days (Figure 5C).

### 2.8. Effects of Salt Stress on Endogenous Hormones 

**Figure 6:**
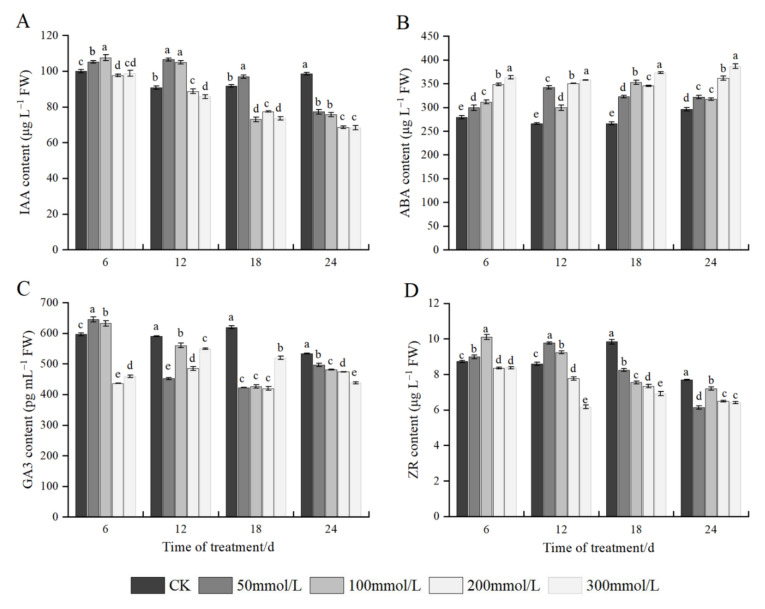
Effect of salt stress on endogenous hormones of *J. microcarpa* L. seedlings. (**A**) Indole-3-acetic acid (IAA), (**B**) abscisic acid (ABA), (**C**) gibberellic acid 3 (GA3), and (**D**) zeatin riboside (ZR) of the leaves. Error bars indicate the SD of treatment means. Different letters on the bars indicate significant differences among the treatments (*p* < 0.05).

As shown in Figure 6A, different NaCl treatment conditions significantly affected the content of IAA in *J. microcarpa* L. leaves. At 6 days of stress, the IAA content increased at first and subsequently decreased with an increase in salt concentration, and the highest IAA content was found at 100 mmol/L. At 12 days of stress, the IAA content in the leaves showed an increase followed by a decrease, in which the IAA content at 50 mmol/L and 100 mmol/L were not significantly different, and both were significantly higher than the control, whereas the IAA contents of other salt treatments were slightly lower than the control. At 18 days of stress, the IAA content increased at 50 mmol/L treatment compared with the control and significantly decreased under the other salt treatments. At 24 days of stress, the IAA content showed a decreasing trend, and the IAA content under each salt treatment content was significantly lower than that of the control. Under the same salt treatment, the IAA content showed an overall decreasing trend with an increase in the stress time. It showed that higher concentration and prolonged salt stress treatment significantly reduced the IAA content in the leaves.

Changes in the ABA content were significantly different among different concentrations of NaCl treatment. Under the same treatment time, the ABA content in the leaves generally showed an increasing trend with an increase in the salt concentration. In addition, the ABA content under each salt treatment was significantly different compared with the control. At 50 and 100 mmol/L concentrations, the ABA content increased at first and then decreased with the prolonged stress time. At 200 and 300 mmol/L concentrations, the ABA content increased with prolonged stress time, but the rate of increase was delayed in the later stages of treatment (Figure 6B).

Different NaCl treatments exerted significant effects on the GA3 content in the leaves. At 6 days of salt stress, with an increase in salt concentration, the GA3 content in the leaves increased and then decreased, and the highest GA3 content was found under 50 mmol/L salt treatment; the GA3 content was significantly decreased at 200 and 300 mmol/L compared with the control. Under the other three treatment times, the GA3 content showed an overall decreasing trend, and the difference was significant compared with the control. Under the same salt treatment, the GA3 content at 50 and 100 mmol/L salt treatment showed a decreasing trend with an extension of the stress time, and the GA3 content under 200 and 300 mmol/L increased at first and subsequently decreased, with unstable changes (Figure 6C).

Different NaCl treatment conditions exerted significant effects on the ZR content in the leaves. At 6 or 12 days of salt treatment, the contents of ZR and IAA showed similar responses. At 18 days of salt stress, the ZR content in the leaves showed a decreasing trend with an increase in salt concentration, and the differences among treatments were significant. At 24 days of salt stress, the ZR content decreased with an increase in salt concentration, and the ZR content in the seedlings at 200 and 300 mmol/L NaCl concentrations was significantly lower than that in the control, but the ZR content was not significantly different between the two treatments. Under the same salt treatment, the ZR content showed an overall decreasing trend an extension of stress time (Figure 6D).

### 2.9. Correlation Analysis

**Figure 7:**
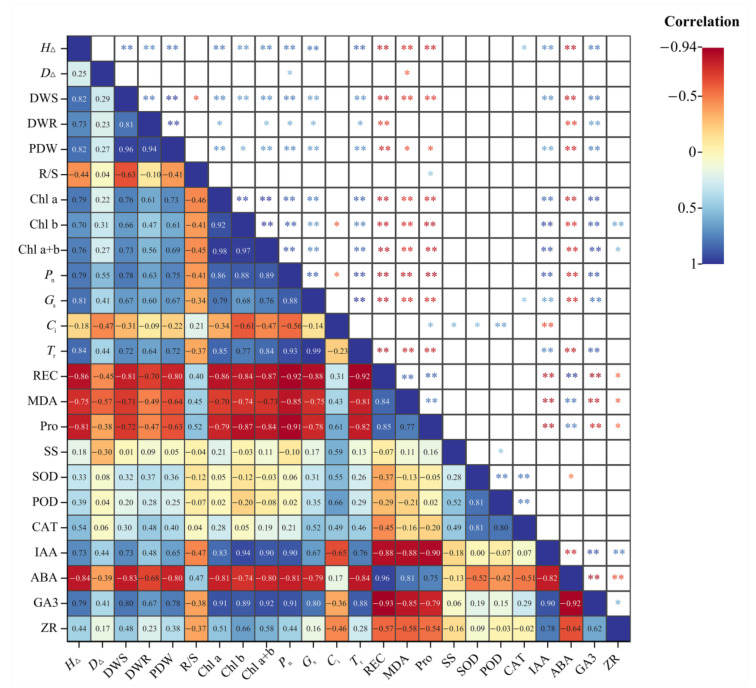
The correlation heatmap of every single index. * means significantly correlation, *p* < 0.05; ** means extremely significantly correlation, *p* < 0.01. Abbreviations of the corresponding indicators are shown in Figure 1, Figure 2, Figure 3, Figure 4, Figure 5 and Figure 6.

Correlation analysis was performed on the growth and final physiological indexes of *J. microcarpa* L. seedlings under salt stress (Figure 7). Results showed that *H*_Δ_, DWS, and PDW were positively correlated with Chl a + b, *P*_n_, *T*_r_, IAA, and GA3, but negatively correlated with REC, MDA, and ABA. The chlorophyll content was significantly correlated with photosynthetic parameters, and negatively correlated with REC, MDA, and Pro. MDA is a product of membrane lipid peroxidation, and REC reflects the cell membrane permeability, REC was highly significantly correlated with MDA. In addition, we observed a highly significant negative correlation between ABA and IAA, GA3, and ZR. In general, growth indicators are closely linked to changes in physiological indicators. When plants are subjected to salt stress, growth decreases while regulating the content of different substances in the plants to maintain normal functions.

## 3. Materials and Methods

### 3.1. Plant Materials and Growth Conditions

The current-year seedlings of *J. microcarpa* L. were used as experimental materials. On 2 January 2021, seeds of uniform size and with no infestation with pests and diseases were selected and placed in a 60 L plastic bucket filled with a certain volume of clean water (the water surface was higher than the seeds). The seeds were soaked for 7 days, and the water was changed once a day. After the water was completely absorbed, the seeds were stored in sand and stratified to accelerate the germination process. On 9 April 2021, the seeds that had been stored in the sand for 3 months were sown in plastic pots of 18 cm in upper diameter and 25 cm in height with a substrate of peat soil, perlite, and vermiculite (3:1:1 *v*/*v*), one seed per pot. The seeds were cultured in the greenhouse of the Chinese Academy of Forestry, China (latitude 40°0′10″ N, longitude 116°14′38″ E, altitude 61 m). The greenhouse culture conditions included an average temperature of 25 °C, not higher than 30 °C during the day and not lower than 14 °C at night, with a transmittance of 50–60% and an average relative humidity (RH) of 55~85%. On 6 May, a few seeds began to emerge, and by 24 May, the emergence rate reached 80%. The seedlings were managed with normal water and fertilizer during the growth period.

### 3.2. Experimental Design

In early August, the seedlings were cultured for around 2 months, and the average plant height of each seedling was 58 cm, the average ground diameter was 4.5 mm, and the average number of compound leaves was 12. Finally, healthy seedlings with consistent growth were selected for NaCl stress treatment. Five salt gradients (0, 50, 100, 200, and 300 mmol/L) were set, and each gradient was divided into three groups of four plants each, with a total of 60 seedlings. During the treatment, the salt solution was applied once a week at the set NaCl concentration (15 August, 22 August, 28 August, and 3 September), each 300 mL, a total of 1200 mL. To prevent the leakage of salt solution, a suitable-sized tray was placed at the bottom of the pot, and the salt solution leaking out of the tray was poured back into the pot. In the morning of 6, 12, 18, and 24 days after salt stress, that is, the morning of 21 August, 27 August, 2 September, and 8 September, the 1–2 pairs of functional leaves of the upper and middle compound leaves were selected to measure photosynthetic indices and related physiological indexes.

### 3.3. Determination of Morphology and Growth Parameters

Before the salt stress treatment, three seedlings with consistent growth were randomly selected from each group, and their seedling height *H*_0_ (measured with a tape measure, cm) and ground diameter *D*_0_ (measured with a Vernier caliper, mm) were measured. After the experiment, the seedling height *H*_1_ and ground diameter *D*_1_ were measured again, and the increment in seedling height *H*_Δ_ = *H*_1_ − *H*_0_ and that in ground diameter increment *D*_Δ_ = *D*_1_ − *D*_0_ were calculated. At the end of the treatment, the seedlings of each treatment group were divided into the aboveground and underground parts, washed, and dried, and their fresh weight was calculated. Next, these were placed in an oven at 105 °C for 30 min and transferred to 75 °C to dry weight; its dry weight was calculated as plant dry weight (PDW) = dry weight of shoot (DWS) + dry weight of root (DWR), root to shoot ratio (R/S) = dry weight of root (DWR)/dry weight of shoot (DWS). The morphological changes in plants were observed and recorded on the 6th, 12th, 18th, and 24th days after salt stress.

### 3.4. Determination of Physiological and Biochemical Indicators

#### 3.4.1. Determination of the Chlorophyll Content

The leaf pigment content was extracted with 95% (*v*/*v*) ethanol by referring to the method described by Zhu et al. [21]. The absorbance was measured at 665 and 649 nm (A_665_ and A_649_). The contents of chlorophyll a (Chl a), chlorophyll b (Chl b), and total chlorophyll (Chl a+b) were calculated using the following equations.

Chl a (mg/L) = 13.95 A_665_ − 6.88 A_649_

Chl b (mg/L) = 24.96 A_649_ − 7.32 A_665_

Chl a + b (mg/L) = 6.63 A_665_ + 18.08 A_649_

#### 3.4.2. Determination of Photosynthetic Parameters

The photosynthetic rate (*P*_n_), stomatal conductance (*G*_s_), transpiration rate (*T*_r_) and intercellular CO_2_ concentration (*C*_i_) of the first and second pairs of functional leaves in the middle and upper compound leaves were measured using a Li-6400 photosynthesis apparatus from 9:00 to 11:00 a.m. on a sunny day. The concentration of CO_2_ was set to 400 μmol mol^−1^, and the light intensity was 1200 μmol m^−2^ s^−1^. A standard leaf chamber was used, the open gas path was adopted, and the flow rate was set to 500 μmol s^−1^.

#### 3.4.3. Determination of Relative Electrical Conductivity

Relative electrical conductivity (REC) was measured using a DDS-11C conductivity meter and assessed according to the method described by Ghalati et al. [22]. The first two pairs of functional leaves of the upper and middle compound leaves of seedlings were collected, washed, and wiped with deionized water, 0.1 g of it was weighed after removing the main veins and leaf margins and incubated in a 100 mL of water bath (40 °C, 30 min), and subsequently, the electrical conductivity R1 was measured. The samples were treated in a boiling water bath for 15 min, and the conductivity R2 was measured again after natural cooling. The REC was used to indicate the cell membrane permeability. The calculation formula used was as follows:

REC (%) = R1/R2 × 100

#### 3.4.4. Determination of Malondialdehyde Content

The malondialdehyde (MDA) content was determined using the thiobarbituric acid (TBA) method [23]. Briefly, fresh leaves were weighed and extracted in 5 mL of 5% (*w*/*v*) trichloroacetic acid (TCA) solution. The supernatant was centrifuged at 10,000 r/min at 4 °C for 10 min. To the supernatant obtained, 2 mL of the TBA solution was added, mixed, and boiled in a water bath for 30 min, rapidly cooled, and centrifuged. The absorbance of the supernatant was read at 450, 532, and 600 nm. The MDA content was calculated as follows:

C = 6.45 × (A_532_ − A_600_) − 0.56 × A_450_

MDA content (μmol/g FW) = C × V_2_ × V/(m × V_1_ × 1000)

where C represents MDA concentration in the extract (μmol/L); V represents the total volume of extract (mL); V_1_ represents the volume of extracted liquid reacting with TBA (mL); V_2_ represents the total volume of extract and TBA reaction solution (mL); m represents the fresh weight of the samples (g).

#### 3.4.5. Determination of Proline Content

The content of proline (Pro) was determined according to the ninhydrin reaction method [24]. In total, 0.1 g of samples were homogenized in 2.5 mL of 3% sulfosalicylic acid solution, and the homogenate was centrifuged at 10,000 rpm for 5 min. The extracted solution (2 mL) was treated with 2 mL of acid ninhydrin and 2 mL of glacial acetic acid and heated for 30 min at 100 °C. Next, 4 mL of methylbenzene was added to the solution after cooling to extract the mixture. Using methylbenzene as the blank control, the absorbance values were recorded using the UV–Vis spectrophotometer (Beijing Purkinje General Instrument Co., Ltd., China) at 520 nm.

#### 3.4.6. Determination of Soluble Sugar Content

The content of soluble sugar (SS) was determined by anthrone colorimetry [25,26]. Samples were extracted with 5 mL of distilled water at 100 °C for 30 min, after which the supernatant was collected. Next, 0.5 mL of the sample extract was placed in a test tube, 1.5 mL of distilled water, 0.5 mL of anthrone reagent (1 g anthrone and 50 mL ethyl acetate), and 5 mL of concentrated sulfuric acid were added, and the solution was shaken thoroughly. The test tube was immediately placed in boiling water for 1 min, removed, and cooled to room temperature naturally. The SS content was analyzed through UV–Vis spectrophotometer (Beijing Purkinje General Instrument Co., Ltd., Beijing, China) at 630 nm.

#### 3.4.7. Determination of Activity of Antioxidant Enzymes

Antioxidant enzymes were extracted by the method of Khalid et al. [27] with slight modifications. Briefly, 0.3 g of the sample was weighed and 3 mL of sodium phosphate buffer (pH 7.8) was added. Next, it was centrifuged at 10,000 r/min at 4 °C for 10 min, and the supernatant was taken as the enzyme extraction solution. The supernatant was collected and used to determine the superoxide dismutase (SOD) and peroxidase (POD) activities.

The SOD activity was determined according to the method described by Liu et al. [26] and Khalid et al. [27], with certain modifications. A total of 0.05 mL of the enzyme extract was taken in a test tube, following which 1.5 mL of the phosphate buffer (pH 7.8), 0.3 mL of methionine, 0.3 mL of NBT, 0.3 mL of EDTA-Na2, 0.3 mL of riboflavin, and 0.25 mL of distilled water were added in order, totaling 3 mL. Among these, the same volume of phosphate buffer was used to replace the enzyme extract for the blank control group. The tubes were placed under 4000 Lx fluorescent lamps for 30 min. After the reaction was over, the lamps were turned off, and the tubes were incubated in the dark for analyses. The SOD activity was determined by monitoring the decrease in the absorbance (560 nm) of the nitroblue tetrazolium (NBT) by the enzyme. The SOD activity was calculated as U/g FW (unit of enzyme activity per gram of fresh weight).

The POD activity was determined using the guaiacol method [10,28]. Briefly, 50 mL of PBS (0.2 mol/L, pH 6.0) was added to 28 μL of guaiacol. The reaction solution was obtained by heating and stirring to dissolve; after cooling, 19 μL of H_2_O_2_ was added to the reaction solution. The mixture was afterward stored in a refrigerator for further use. Next, 3 mL of the reaction solution was taken, and 40 μL of the enzyme solution was added. PBS served as the blank, and the absorbance values of solutions were measured at 470 nm. One unit of the POD activity was expressed as the change in the absorbance per min.

The catalase (CAT) activity was measured using a CAT assay kit (Solarbio, Beijing, China). Briefly, 0.1 g of the material was homogenized in an ice bath by adding 1 mL of the extraction solution and centrifuged at 8000× *g* for 10 min at 4 °C. The supernatant was removed and placed on ice for measurement. The microplate reader was preheated for over 30 min, the wavelength was adjusted to 240 nm, and the distilled water was transferred to zero. Next, 10 μL of the sample and 190 μL of the working liquid were added to a 96-well plate and immediately mixed; the initial absorption values A1 under 240 nm and A2 after 1 min were recorded and calculated ΔA = A1 − A2. The catalytic degradation of 1 μmol H_2_O_2_ per minute per g of material in the reaction system was defined as an enzyme viability unit.

#### 3.4.8. Determination of Endogenous Hormones

A certain amount of plant leaves was weighed and homogenized in an ice bath with PBS (0.01 mol/L, pH 7.2–7.4). The weight of the sample weighed is not less than 50 mg; generally, 1 g is taken as the benchmark. The proportion of homogenate is chosen to be 10%, which is equivalent to 1 g of tissue plus 9 mL of solution for homogenization. The homogenate was centrifuged for 20 min at 2000–3000 rpm, and then the supernatant was taken to be tested.

Indole-3-acetic acid (IAA), abscisic acid (ABA), gibberellic acid 3 (GA3), and zeatin riboside (ZR) were detected by enzyme-linked immunosorbent assay (ELISA), following the manufacturer’s instructions (Jiangsu Jingmei Biological Technology Co., Ltd., Yancheng, China).

### 3.5. Statistical Analysis

After the data were preprocessed with Microsoft Excel 2019, version, 2207 (Microsoft Corp., Washington, DC, USA), ANOVA analysis and Duncan’s multiple comparisons were performed using IBM SPSS Statistics, version 23 (International Business Machines Corp., Armonk, NY, USA) *p* < 0.05), analyzed with Origin 2018, and plotted the changes in indicators. SPSS 23.0 was used to analyze the growth indicators and final physiological indicators of *J. microcarpa* L. under salt stress by Pearson’s correlation analysis. The correlation between each indicator was compared, and the correlation heat map was drawn using ChiPlot (https://www.chiplot.online/) (accessed on 7 August 2022).

## 4. Discussion

The morphological characteristics of plants visually reflect their growth status, which is an important index of plant salt tolerance [8,29]. The salt tolerance of two pistachio rootstock varieties by Rahneshan et al. [24] and four kiwifruit genotypes by Abid et al. [8] showed a series of salt damage symptoms such as yellowing, withering, and abscission of leaves to varying degrees under salt stress. When plants were subjected to external stresses, their physiological indexes will change, thus characterized in their external morphology so that morphological characteristics are the most intuitive manifestations of plants under environmental stress. During the early stage of stress, no obvious symptoms of salt damage were observed in *J. microcarpa* L. With an extension of stress time, the leaves of different treatments showed yellowing, withering, curling, and shedding due to a decrease in the content of Chl a, Chl b, and Chl a+b—the most important pigments synthesized by plants through photosynthesis. At 12 days of stress, salt damage symptoms already appeared at 200 and 300 mmol/L concentrations, and the salt damage symptoms first appeared on the lower old leaves of the plant. At 100 mmol/L and below, salt damage symptoms appeared later and to a lesser extent, whereas above 100 mmol/L, salt damage symptoms were significant and plants were more severely affected.

The changes in plant growth indexes and biomass are the comprehensive manifestation of plant response to salt stress [30,31]. Our study showed that with an increase in salt concentration, *H*_Δ_, *D*_Δ_, DWS, and DWR of *J. microcarpa* L. seedlings decreased, indicating that salt stress inhibited the growth and accumulation of biomass. Although salt stress at 50 mmol/L and 100 mmol/L did not have a significant effect on seedlings, it exerted an inhibitory effect to a certain extent. Moreover, salt stress of 200 mmol/L and above significantly inhibited the growth of seedlings. Ion toxicity and nutrient imbalance are among the most important reasons for reduced plant growth under high salinity [32]. In addition, soluble salts in the soil increase the osmotic pressure and decrease the total soil water potential, thus reducing water uptake by the roots, which also ultimately leads to a reduction in plant growth parameters [33]. In the present study, we measured photosynthetic rate and stomatal conductance, which showed a decreasing trend with the deepening of salt stress. It has been shown that under stress conditions, plants close their stomata to prevent water loss by transpiration, and this mechanism limits the assimilation of CO_2_, which slows down the photosynthetic process and limits plant growth [34]. Induced stomatal closure under salt stress is a physiological mechanism for plants to reduce or avoid salt injury. One of the reasons for the decrease in growth parameters in this study could be due to the closure of the stomatal, reduced carbon dioxide emissions and photosynthetic efficiency [33,35]. The literature has reported that salinity could increase the R/S ratio because salinity can rapidly inhibit shoot growth while maintaining root growth [36]. Consistent with this, although salt stress inhibited the growth of the aboveground and underground parts of *J. microcarpa* L. seedlings, the R/S ratio increased, indicating that the aboveground part was more severely affected than the underground part. This plant response to improve the R/S ratio by changing the biomass allocation patterns is one of the primary strategies of plants to adapt to stresses [37].

Chlorophyll, as the most basic photosynthetic pigment in plants, is involved in the absorption, transmission, and transformation of light energy, and its content is an important indicator reflecting the strength of photosynthesis [24,38]. With an increase in the salt concentration and prolonged stress time, Chl a, Chl b, and Chl a+b contents in the seedings showed an overall decreasing trend, which could be attributed to the damage to the chloroplast structure caused by salt stress, and the activity of chloroplast enzymes increased, which promoted the breakdown of photosynthetic pigments [5,21]. The decrease in chlorophyll concentration indicated oxidative damage to chloroplasts, which ultimately led to a significant decrease in CO_2_ assimilation rate and non-stomatal restriction of photosynthesis [12]. Our results were similar to the findings of Dichala et al. [2] that chlorophyll was reduced as NaCl concentrations increased.

Ordinarily, photosynthesis has been recognized as a source of material and energy for their growth. Many studies have concluded that the reduction in photosynthetic rate (*P*_n_) under NaCl stress is the result of several physiological responses, including reductions in stomatal conductance (*G*_s_), transpiration rate (*T*_r_), chlorophyll, and carotenoid contents, or a combination of these parameters [39,40]. In general, the first physiological response to salt stress is to avoid water loss through transpiration, which is achieved by a decrease in *G*_s_ value due to stomatal closure [40]. At the early stage of salt stress (6 d and 12 d), *P*_n_, *T*_r_, *C*_i_, and *G*_s_ of *J. microcarpa* L. leaves decreased with an increase in salt concentration, indicating that it could be attributed to stomatal closure; this is similar to the findings of Lu et al. [40] and Yuan et al. [41]. Notably, with the prolongation of salt stress (24 d), *P*_n_, *G*_s_, and *T*_r_ decreased, while *C*_i_ increased with an increase in salt concentration. A similar result was found in salt-stressed melon as well [39]. This change in photosynthetic indexes at high salt concentrations may be caused by nonstomatal limiting factors [40,42]. After salt stress, *J. microcarpa* L. is affected by both stomatal and non-stomatal limitation, which reduces photosynthesis, and the dynamic changes between stomatal and non-stomatal limitation change with salt concentration and stress time.

The membrane system of plants is the first site to sense the damage under stress conditions such as salt stress. Damage to the cell membrane system is manifested as membrane lipid oxidation reactions, resulting in increased cell membrane permeability and elevated REC. MDA is one of the products of membrane lipid peroxidation in plants, such that REC and MDA are two important physiological indexes of the extent of plant damage under stress conditions [12,21]. Studies have shown that MDA affects the structure of thylakoid membrane and leads to the degradation of chlorophyll, thus affecting photosynthesis in plants [43]. At the same time, MDA, a product of membrane lipid peroxidation, also has a feedback effect on the antioxidant protection system of cells and affects its antioxidant enzyme activity [22]. Yang et al. [44,45] monitored MDA content in leaves as an indicator of abiotic stress pressure on *J. regia*. In this study, under low concentration of salt stress (50–100 mmol/L NaCl), MDA content in *J. macrocarpa* L. seedlings increased slowly, which might be due to the low concentration of salt stress promoting the activity of antioxidant enzymes, which reduced the damage to the cell membrane system. However, with the increase in salt concentration and the prolongation of stress time, MDA content increased significantly. The variation of REC is similar to MDA. This indicates that the high concentration of NaCl suggests the seedlings already have cell membrane lipid peroxidation, the function of the antioxidant system of the cell membrane is weakened, and the degree of cell membrane damage is increased, which affects the physiological metabolic process of *J. macrocarpa* L.

Under salt stress, plants can synthesize and accumulate organic osmolytes in excess amounts to ensure plant tolerance against osmotic stresses [8]. Previous studies have shown that proline is a compatible osmolyte accumulated by many plants and microorganisms in response to osmotic stress caused by salinity and drought [29,46]. The accumulation of proline helps in membrane stability, which in turn mitigates the adverse effect of NaCl on cell membrane disruption [47]. In this study, the overall trend of Pro content in *J. microcarpa* L. seedlings increased with an increase in salt concentration and prolonged stress time, which is consistent with the results of other studies on environmental stresses such as salinity stress on citrus rootstocks [47], water and salinity stress on late-bearing walnut [48] and acid stress on five citrus rootstocks [21]. Under prolonged high salt stress, Pro content increased significantly, indicating that although high salt stress accelerated the peroxidation of membrane lipids, promoted the accumulation of peroxidation products of MDA, and cell membrane damage, *J. microcarpa* L. could alleviate the osmotic stress to a certain extent by increasing Pro content. Recent studies have shown that SS play a key role in plants’ osmotic adjustment, such as maintaining cell turgor, water uptake and transport under stressful conditions [48,49]. In this study, with the increase in the salt concentration and stress time, the SS content increased at first, followed by a decrease. Under the conditions of pre- and mid-salt stress and medium- and low-concentration NaCl stress, SS, as an osmotic adjustment substance, maintained the proper osmotic potential level by increasing its own content, slowing down cell water loss, and reducing stress injury. However, under prolonged and relatively high NaCl stress conditions, the SS in plants underwent decomposition, and the level of decomposition was greater than the level of synthesis, thus reducing the total amount of SS, weakening plant resistance, and aggravating injury, thus reducing salt stress resistance.

Under normal conditions, the contents of superoxide anion radicals (O^2−^), singlet oxygen (O_2_), hydrogen peroxide (H_2_O_2_), and hydroxyl radicals (OH^−^) in plant cells are very low, and the intracellular ROS production and scavenging are in dynamic equilibrium [21]. However, stress conditions disrupt this equilibrium, and ROS in plants accumulate in large amounts, and disrupted normal metabolism through oxidative damage to lipids, proteins and nucleic acids in the absence of any protective mechanism [5]. Therefore, under stress conditions, a series of antioxidant enzymes (such as CAT, SOD, and POD) are generated following the accumulation of ROS in plants to eliminate the excess ROS. The equilibrium between the effective production and elimination of ROS can be used as secondary messengers to abiotic stresses, and could be used to evaluate the tolerance of plants [9,21]. The results showed that with an increase in salt concentration and the extension of stress time, the SOD and CAT activities showed an overall trend of the first increase, followed by a decrease. During the early stage of stress, the enzyme activity increased greatly compared with the control with an increase in salt concentration. During the late stage of stress, the increase in enzyme activities was small. During the late stage of stress, with a decrease in enzyme activities, SOD and CAT activities were still higher than the control at concentrations below 200 mmol/L, and on the 28th day, the enzyme activities were lower than the control at 200 and 300 mmol/L. During the early stage of stress, the POD activity increased with an increase in salt concentration. During the late stage of stress, the POD activity increased, followed by a decrease with an increase in the salt concentration, reaching a peak at 100 mmol/L. On the 28th day, the enzyme activity was lower than that of the control at 300 mmol/L. The above results showed that under salt stress, the antioxidant capacity of *J. microcarpa* L. was enhanced to scavenge excessive ROS and reduce the damage of ROS on cell membranes, and within a certain range, the activities of SOD, POD, and CAT were higher, and each oxidative enzyme could prevent the damage caused by harmful substances such as reactive oxygen species. However, prolonged or high salt stress exceeded the tolerance range of *J. microcarpa* L. seedings, decreasing the ability to scavenge peroxide radicals, and causing an imbalance in the plant’s own metabolism and a decrease in enzyme activity, and the growth of *J. microcarpa* L. became worse. These findings are consistent with those of Wang et al. [23] and Liu Hao et al. [3].

Plant hormones, also known as phytohormones, are small chemicals that play a crucial role in plant growth and development [15]. Under stress conditions, plants can control growth rhythms and metabolic activities by regulating the content of hormones in the bodies to ensure normal maintenance of physiological functions and successful adaptation to the external stressful environment [15,50]. The study by Sachs [51] had reported that the allocation of dry matter to plant tissues is essentially controlled by plant hormones. In the present study, both the increase of ABA and decrease of GA3 in *J. macrocarpa* L. seedling leaves could lead to the inhibition of leaf growth under t salt stress. This was also indicated by the significant correlation between ABA, GA3 and biomass. Consistent with the results of the present study, a relationship between ABA and biomass regulation under salt stress was also shown in cotton plants [7] and tomato plants [52]. Under abiotic stress such as drought and salt, ABA rapidly accumulate in the plants to regulate functions such as the closure of stomata, reduction of transpiration rate, and water loss, consequently reducing the damage caused by salt stress [16,53,54]. In this study, the IAA content in the leaves increased at first and subsequently decreased with an increase in stress concentration during the early stage of stress, whereas the IAA content showed a downward trend with an extension in the stress time. This indicates that low salt stress does not pose a serious threat to the growth of plants. In the present study, ABA was increased under salt stress and there was a significant negative correlation between *G*_s_ and ABA. This result indicates ABA could be involved in *G*_s_ regulation in *J. macrocarpa* L. in response to salinity. The contents of GA3 and ZR showed an overall decreasing trend with the deepening of salt stress, and there was a significant positive correlation between GA3, IAA and Gs. Previous studies have demonstrated that, in addition to ABA, other phytohormones, including auxin, gibberellin, cytokinin and ethylene, could also be involved in stomatal regulation under stress [7,55]. It has also been suggested that the changed phytohormonal balance might be a factor directly involved in stomatal regulation [56]. Ma et al. [7] also indicated that both leaf ABA and leaf GA3 could be involved in *G*_s_ regulation. These results indicating that both ABA, GA3 and IAA might be involved in the regulation of *G*_s_ in *J. macrocarpa* L. Nevertheless, to date it remains largely elusive about the interaction effects of phytohormones such as ABA, IAA, GA3 and ZR on stomatal movements when plants exposed to salt stress, and further investigations are needed. In general, under salt stress conditions, *J. microcarpa* L. promotes the production of ABA while inhibiting the synthesis of IAA, GA3, and ZR through coordination of multiple hormones, thereby comprehensively regulating the growth and development of plants and their physiological and biochemical responses after salt stress.

## 5. Conclusions

All physiological indexes of *J. microcarpa* L. showed a certain response to salt stress. Under salt stress, *J. macrocarpa* L. seedlings alleviate osmotic stress by increasing the content of osmotic adjustment substances; maintaining the balance of reactive oxygen species in the body and reducing the oxidative damage by increasing the activities of antioxidant enzymes. And regulating their physiological metabolism and growth through mutual coordination of multiple hormones, thereby, enhancing their salt tolerance. The analysis of the growth status and physiological and biochemical indexes revealed that *J. microcarpa* L. seedings displayed a certain level of salt tolerance, and it showed tolerance of salt stress with 100 mmol/L NaCl. However, under high salt stress ranging from 200 to 300 mmol/L, the growth of *J. microcarpa* L. was severely affected and inhibited.

## Figures and Tables

**Table 1:** Symptoms levels of salt injury level.

Grade	Salt Stress Symptoms
0	No symptoms of salt stress damage.
1	A few tips and margins of the lower old leaves turn yellow and wither.
2	Some tips and margins of the lower old leaves turn yellow and wither.
3	Some tips and margins of the lower old leaves were withered, and the withered and curled number of the middle and upper leaves increased.
4	Most leaves are withered and curled, and some compound leaves have withered petioles.
5	Most leaves are withered and curled, accompanied by falling; the lower compound leaves and total petioles are withered, accompanied by a few fallen off.
6	Most leaves are withered, curled, and fallen off, and most compound leaves and total petioles are withered and fallen off.

**Table 2:** Leaf salt injury classification of *J. microcarpa* L.

NaCl /(mmol·L^−1^)	Stress Symptom Grade			
	6 d	12 d	18 d	24 d
50	0	0	1	3
100	0	0	2	3
200	0	2	3	5
300	0	2	5	6

**Table 3:** Growth indexes of the seedlings of *J. microcarpa* L. under salt stress. The data are presented as treatment mean ± SD. Different English letters in the same column indicate a significant difference at the level of 0.05.

NaCl /(mmol·L^−1^)	*H*_Δ_/cm	*D*_Δ_/mm	DWS/g	DWR/g	PDW/g	R/S
0	1.133 ± 0.058 a	0.907 ± 0.291 a	8.187 ± 0.973 a	7.890 ± 1.068 a	16.077 ± 1.842 a	0.966 ± 0.102 a
50	1.067 ± 0.153 a	0.808 ± 0.384 a	7.083 ± 1.320 ab	7.950 ± 1.541 a	15.033 ± 2.838 ab	1.122 ± 0.049 a
100	0.933 ± 0.058 a	0.598 ± 0.140 a	6.437 ± 0.883 ab	7.010 ± 0.879 ab	13.477 ± 1.746 ab	1.091 ± 0.044 a
200	0.633 ± 0.208 b	0.588 ± 0.122 a	4.970 ± 1.537 bc	6.510 ± 0.518 ab	11.480 ± 1.686 bc	1.410 ± 0.487 a
300	0.600 ± 0.173 b	0.582 ± 0.304 a	4.053 ± 1.220 c	4.883 ± 1.727 b	8.937 ± 2.600 c	1.247 ± 0.449 a

*H*_Δ_, plant height increment; *D*_Δ_, ground diameter increment; DWS, dry weight of shoot; DWR, dry weight of root; PDW, plant dry weight; R/S, the ratio of root to shoot.

## Data Availability

Not applicable.
